# Active learning and decision making: an introduction to the collection

**DOI:** 10.12688/f1000research.5757.2

**Published:** 2015-01-15

**Authors:** Jacqueline Gottlieb, Manuel Lopes, Pierre-Yves Oudeyer

**Affiliations:** 1 Department of Neuroscience, The Kavli Institute for Brain Science, Columbia University, New York, NY, USA; 2 INRIA, Bordeaux Sud-Ouest, France

## Abstract

The importance of exploratory behaviors by which agents actively sample information has been long appreciated in a wide range of disciplines ranging from machine and robot learning to neuroscience and psychology.  Given the complexity of these behaviors, progress in understanding them will require a confluence of ideas from these multiple fields.  This collection of articles in *F1000Research* aims to provide a home for a broad range of studies addressing this topic, including full length research articles, brief communications, single figure studies, and review/opinion articles, and studies using computational, behavioral or neural approaches.  Here, we provide an introduction to the collection which we hope will grow and become a valuable resource for the researchers exploring this topic.

## Editorial

Most of our decisions are made under uncertainty, and many of our actions are geared toward reducing this uncertainty. Information seeking actions take many shapes and forms that span the gamut of cognitive function. At one end of the range are simple orienting acts whereby we use our sensory receptors to sample task-relevant information – such as *looking* at a relevant stimulus or *listening* for a relevant sound. At the other end are elaborate behaviors such as scientific research, which systematically search for information over extended time scales. And at an intermediate level there are exploration/exploitation tradeoffs, whereby we may temporarily forego a valuable action in order to learn about more uncertain but potentially more lucrative paths.

Understanding how the brain regulates its information seeking behaviors is significant from both basic science and applied perspectives. It is important for understanding attention, which is a crucial information selection mechanism and is implicated in a range of psychiatric disorders ^1^, for understanding the active control of learning and memory – how a neural system selects and organizes its own learning experiences ^4^ and determines which ones will leave a lasting trace ^2^, and for understanding curiosity and embodied exploration during development and adulthood ^3, 6^. From a practical standpoint, such an understanding can spur improvements in education and computational methods for guiding efficient robotic exploration ^6, 7^.

Addressing these questions requires us to tackle a number of difficult questions ^4^. These questions include how rewards and information seeking shape cognitive mechanisms of learning, memory and attention ^5^, how information seeking shapes exploratory behavior in situated and embodied organisms ^6^, how subjects build explanatory models of their environment and use these models to constrain the sampling of additional information, how the brain generates the intrinsic motivation to seek information when physical rewards are absent or unknown, and how intrinsic and extrinsic rewards interact in driving behavior. The goal of this collection is to provide a home for papers on these and related topics, with the goal of spurring interest and debate in this research field.
